# Evaluation of microhardness, monomer conversion, and antibacterial properties of an experimental pulp-capping material containing collagen–hydroxyapatite nanocomposite and/or chlorhexidine

**DOI:** 10.1007/s10266-025-01113-5

**Published:** 2025-04-28

**Authors:** Hacer Balkaya, Sezer Demirbuğa, Fatih Duman, Ahmet Ceylan, Ömer Aydın

**Affiliations:** 1 Department of Restorative Dentistry, Faculty of Dentistry, Erciyes University, 38039 Kayseri, Turkey; 2 Faculty of Science, Biology, Hydrobiology, Erciyes University, 38039 Kayseri, Turkey; 3 Faculty of Pharmacy, Pharmaceutical Technology, Pharmaceutical Biotechnology, Erciyes University, 38039 Kayseri, Turkey; 4 Faculty of Engineering, Biomedical Engineering, Erciyes University, 38039 Kayseri, Turkey

**Keywords:** Hydroxyapatite, Collagen, Collagen–hydroxyapatite nanocomposite, Pulp capping, Experimental pulp-capping material

## Abstract

This study aimed to develop and characterize an experimental pulp-capping material incorporating collagen–hydroxyapatite nanocomposite (cHAP) derived from fish scales and chlorhexidine (CHX) as an antimicrobial agent. The synthesized cHAP was characterized using XRD, FT-IR, EDX, FE-SEM, and BET analyses. The nanocomposite and/or CHX were loaded onto a commercially available resin-based pulp-capping material (TheraCal LC). Experimental groups were defined as Control group, 1% cHAP (cHAP1), 5% cHAP (cHAP5), 5% chlorhexidine (CHX), and 2.5% CHX + 2.5% cHAP (cHAP-CHX). Standardized samples (6 mm diameter, 1 mm height) were prepared from experimental pulp-capping materials using a Teflon mold for subsequent analyses. Microhardness, monomer conversion, and antibacterial activity of the materials were investigated following SEM–EDX, XRD, and FT-IR analyses. Data were analyzed using one-way analysis of variance (ANOVA) and Tukey’s post hoc test, with a significance level of *p* < 0.05. The cHAP and experimental pulp-capping materials were successfully characterized. CHX incorporation decreased microhardness significantly (*p* < 0.05), whereas cHAP-containing groups showed no significant differences compared to the control (*p* > 0.05). The degree of monomer conversion was unaffected by the addition of cHAP or CHX individually (*p* > 0.05), but a significant increase was observed when both CHX and cHAP were added (*p* < 0.05). Antibacterial testing revealed that *E. faecalis* was the most sensitive strain against the tested pulp-capping materials, with the cHAP-CHX group exhibiting the highest antimicrobial activity. The CHX and cHAP-CHX groups demonstrated antimicrobial activity against both *E. faecalis* and *S. mutans*, while the cHAP5 group was effective only against *E. faecalis*. The control group showed no antimicrobial activity against either strain. The addition of cHAP and CHX to the pulp-capping materials enhanced monomer conversion. Pulp-capping materials containing CHX and cHAP-CHX were particularly effective against *E. faecalis* and *S. mutans.* The integration of cHAP and CHX into the experimental resin-based pulp-capping materials offers a promising strategy for improving antibacterial activity and biocompatibility. This combination may serve as a potential candidate for enhancing pulp-capping procedures in clinical practice.

## Introduction

Vital pulp therapies, including indirect and direct capping, as well as pulpotomy procedures, are widely used to manage reversible pulpitis. These treatments aim to preserve the health of exposed pulp tissue and stimulate the natural repair mechanisms of the pulp. This is achieved by applying biocompatible pulp-capping materials to the site of pulpal exposure, which protect the pulp from further stress caused by dental procedures, the toxicity of restorative materials, and bacterial infiltration resulting from microleakage [1]. Such materials directly or indirectly induce the deposition of tertiary dentin, replacing tissue lost due to carious lesions or trauma [2, 3]. Ideally, pulp-capping materials should exhibit properties, such as excellent sealing ability, biocompatibility, bioactivity, antibacterial activity, bonding to dentin, insolubility in tissue fluids, affordability, and ease of application [4, 5]. While current pulp-capping materials exhibit significant advantages in promoting pulp healing and dentin formation, there is ongoing research to enhance their performance. Efforts focus on improving additional properties, such as bioactivity, antibacterial efficacy, and handling characteristics, to further optimize pulp-capping outcomes.

Pulp-capping materials include calcium hydroxide (CH), mineral trioxide aggregate (MTA), and calcium silicate-based materials. Calcium silicate-based materials, such as Biodentine and TheraCal, are more recently introduced alternatives that offer enhanced biocompatibility and shorter setting times (e.g., 12 min for Biodentin) compared to MTA [6, 7]. Despite their faster setting times, calcium silicate-based materials may still delay certain restorative procedures. Resin-modified calcium silicate cements, which integrate resin into calcium silicate, are considered promising due to their improved handling and faster setting times, offering clinical convenience. However, they release unpolymerized monomers that can be cytotoxic to pulp cells. This toxicity may occur directly, as monomers diffuse into the pulp tissue, or indirectly, through dentinal tubules [1, 8]. To minimize these risks, achieving a high degree of polymerization is critical to reduce monomer release and diffusion [8].

Dentin is primarily composed of inorganic components (approximately 70%), along with 20% organic material and 10% water. The organic matrix is predominantly made up of type I collagen (90%), alongside non-collagenous proteins (6.7%), citric acid (0.9%), proteoglycans, and lipids (0.2%). The inorganic portion mainly consists of apatite, predominantly hydroxyapatite, with smaller amounts of calcium carbonate, amorphous phosphate, magnesium ions, and trace elements [9]. Hydroxyapatite (HAp) and type I collagen are frequently used in bone tissue engineering due to their bioactivity and biocompatibility [10–12]. Moreover, it is believed that the simultaneous presence of collagen and HAp in a composite structure offers significant advantages for new tissue formation [13].

Traditionally, HAp and collagen have been sourced from land animals, such as cattle, sheep, and pigs. However, their use is limited due to concerns about transmission of zoonotic diseases, including foot-and-mouth disease, bovine spongiform encephalopathy, and transmissible spongiform encephalopathy [14, 15]. Additionally, the use of bovine and porcine collagen may conflict with certain religious beliefs [16, 17]. As a result, fish have emerged as an alternative source of HAp and collagen [18]. In particular, fish scales stand out as a natural source of both collagen (mostly Type I) and HAp [19]. Fish scale-derived collagen/ HAp nanocomposite (cHAP) offers several advantages, including being rich in collagen and HAp, biocompatible, cost-effective, safer than mammalian sources, and free from the risk of animal-borne disease transmission [20, 21]. Moreover, using fish-derived materials represents an environmentally friendly approach, repurposing waste from the food industry into valuable biomaterials. In addition, fish-derived scaffolds have several advantages: they are easier to obtain, exhibit low antigenicity, and contain unique amino acid sequences that promote cell adhesion, proliferation, and differentiation [22]. Thus, the proposed material has the potential to be utilized as a scaffold in regenerative treatments.

Chlorhexidine (CHX) is a cationic agent widely used in medical and dental fields for its potent antimicrobial properties. It is effective against a broad spectrum of microorganisms, including Gram-positive and Gram-negative bacteria and fungi [23]. CHX effectively kills a wide range of bacteria, including those that infect dental pulp tissue, making it particularly valuable in endodontic treatments and direct pulp capping. By reducing the microbial load at the site of exposure, CHX helps minimize the risk of infection and inflammation in the pulp tissue. In pulp revascularization therapy, a 2% CHX solution is commonly used as an irrigant [24]. CHX has also been shown to inhibit matrix metalloproteinases (MMPs), enzymes involved in dentin degradation [25]. Moreover, a previous study has shown that CHX, when incorporated into a resin material (with or without hydroxyapatite), continues to release over an extended period, up to 120 days [26]. Thus, incorporating a nanostructure containing collagen–hydroxyapatite and CHX into dental materials could enhance their biological properties.

To date, no studies have investigated a pulp-capping material incorporating cHAP derived from fish scales. Therefore, this study aimed to characterize experimental pulp-capping materials containing cHAP and/or CHX and to evaluate their microhardness, degree of monomer conversion, and antibacterial activity. Specifically, it focused on materials formulated with cHAP extracted from fish scales, either with or without CHX.

The research hypotheses were as follows:

1. Incorporating cHAP into the pulp-capping material, alone or in combination with CHX, will increase the microhardness of the material.
2. Adding cHAP to the pulp-capping material, alone or in combination with CHX, will increase the degree of monomer conversion of the material.
3. Incorporating cHAP, alone or in combination with CHX, will enhance the antibacterial activity of the material.

## Materials and methods

### Fabrication of cHAP nanocomposites

Sea perch (*Dicentrarchus labrax* L.) samples were purchased from a fish market in Kayseri, Turkey. In this study, sea perch scales were used as the primary material. The scales were separated using a fish scale sorting apparatus, and foreign materials (e.g., mucus, algae, and debris) were removed using a soft brush and ultrapure water. Decellularization was performed by treating the scales with 0.2 M NaOH, followed by sonication for 5 min at 40 °C. The scales were then rinsed with ultrapure water until the pH was neutralized. The effectiveness of the decellularization was confirmed via nuclei staining and fluorescence microscopy (ECLIPSE Ni, Nikon, Tokyo, Japan). The decellularized scales were ground into a fine powder using a laboratory grinder. The powdered sample was freeze-dried at −80 °C for 24 h in a freeze dryer (Alpha 2–4 LSCplus, Christ, Osterode, Germany) to remove residual moisture. The freeze-dried powder was stored in a desiccator (Labor Teknik, Istanbul, Turkey) to maintain dryness, eliminating the need for freezer storage. The final cHAP nanocomposite had a hydroxyapatite-to-collagen ratio of 60: 40 by weight.

### Characterization of the cHAP nanocomposites

Field emission scanning electron microscopy (FE-SEM) was used to visualize surface topography and particle structure of the cHAP, energy-dispersive X-ray spectroscopy (EDX) to characterize the elemental composition and mapping, X-ray diffraction (XRD) to examine the crystal structure, Fourier transform infrared spectroscopy (FT-IR) to evaluate molecular bond structures, and Brunauer–Emmett–Teller (BET) analysis to determine the pore size distribution and pore volume.

For FE-SEM analysis, the sample surfaces were coated with a gold–palladium (Au–Pd) alloy at a ratio of 20%-80% for 15 s. The coating was applied in increments of 15 Å every 5 s to achieve a final thickness of 45 Å. The coated surfaces were examined under a field emission scanning electron microscope (26 kV, GeminiSEM 500, Zeiss, Oberkochen, Germany) at magnifications of 2000 × and 20,000x.

### Fabrication and characterization of experimental pulp-capping materials

Chlorhexidine diacetate (CHX), used in combination with cHAP, was procured from Sigma-Aldrich (St. Louis, MO, USA). The nanoparticles were incorporated into a commercially available resin-based pulp-capping material (TheraCal LC; Bisco Inc, Schamburg, IL, USA). The study groups were formed as follows:

1. Control group: A commercially available resin-based pulp-capping material (TheraCal LC),
2. cHAP1: Experimental resin-based pulp-capping material containing 1% cHAP,
3. cHAP5: Experimental resin-based pulp-capping material containing 5% cHAP,
4. CHX: Experimental resin-based pulp-capping material containing 5% chlorhexidine,
5. cHAP-CHX: Experimental resin-based pulp-capping material containing 2.5% cHAP and 2.5% chlorhexidine.

The pulp-capping materials were placed in a Teflon mold and compressed between two glass slides. The materials were then light-cured for 20 s using an LED light device (Valo, 1000 mW/cm^2^, Ultradent Products, South Jordan, UT, USA) to obtain samples with a standard diameter of 6 mm and a height of 1 mm. The output energy of the LED-curing device was measured periodically using a radiometer.

After completing SEM–EDX, XRD, and FT-IR analyses for characterization of the experimental materials, additional tests were performed.

### Vickers microhardness testing

The microhardness of disk-shaped samples (*n* = 6) from each group was measured using a microhardness tester (DuraScan hardness tester, EMCO TEST, Austria). For each sample, the average of three measurements taken from different regions on the upper surface using a diamond indenter was recorded. The two diagonal lengths of the resulting indentation were measured under a microscope at 40 × magnification. The microhardness (VHN) value was calculated using the following formula:

HV = 1.854 × P/d^2^,

where HV is the microhardness in kg/mm^2^, P is the applied load in kgf, and d is the average length of the diagonals in mm.

### Degree of monomer conversion (DC)

The degree of monomer conversion (DC) of the experimental resin-based pulp-capping materials was measured using a Fourier Transform-Infrared Spectrophotometer (Spotlight 400 FT-IR Imaging System, PerkinElmer, Waltham, MA, USA) (*n* = 6). The ATR platform and measurement tip were cleaned with ethanol after each scan. Following an initial reference measurement, 20 additional scans were performed for each sample at a resolution of 4 cm^−1^ within a wavenumber range of 400–4000 cm^−1^.

The DC was calculated by analyzing the absorbance peaks corresponding to the aromatic C = C bonds around 1608 cm^−1^ and the aliphatic C = C bonds around 1638 cm^−1^, using the following formula:

$${\text{DC }}\left( \% \right) \, = \left( {{1} - \frac{{\left( {Absorbance at 1638 cm^{ - 1} /Absorbance at 1608 cm^{ - 1} } \right) after curing}}{{\left( {Absorbance at 1638 cm^{ - 1} /Absorbance at 1608 cm^{ - 1} } \right) before curing}}} \right) \times 100$$

### Antibacterial activity testing

The antimicrobial activities were evaluated against the following microorganisms: *E. faecalis* and *S. mutans*. Prior to antibacterial activity testing, the samples were sterilized under ultraviolet light for 2 h on each side. The antibacterial activity of the materials against *Enterococcus faecalis* (*E. faecalis,* ATCC 29212) and *Streptococcus mutans* (*S. mutans,* ATCC 25175) strains was assessed using the agar diffusion method. Bacteria were cultured on Mueller–Hinton agar (MHA, Bio-Rad Laboratories, France). Bacterial suspensions with a concentration of 6 × 10^5^ colony-forming units (CFU)/mL were prepared from preliminary cultures according to the 0.5 McFArland turbidity standard. 100 μL of bacterial suspension were inoculated and spread evenly on the MHA agar plates. The samples were placed onto the inoculated agar surface under aseptic conditions. After incubating the plates at 37 °C for 24–48 h, the diameters of inhibition zones around the disk-shaped samples were measured in millimeters using Vernier calipers (Mitutoya 0.02 mm 505-646-50, Shock Proof, Kawasaki, Japan). The measurements were obtained in four replicates, and arithmetic averages were calculated for analysis.

### Statistical analysis

Data were analyzed using a SPSS version 22.00 (IBM Inc., Armonk, NY). The Shapiro–Wilk test was conducted to assess the normality of data distribution. One-way analysis of variance (ANOVA) was used to compare differences between the groups in terms of surface hardness, monomer conversion, and antibacterial activity. Additionally, a Tukey post hoc test was conducted for multiple comparisons among the groups. A *p* value of < 0.05 was considered statistically significant for all comparisons.

## Results

### Surface morphology and composition of cHAP nanocomposites

**Fig. 1:**
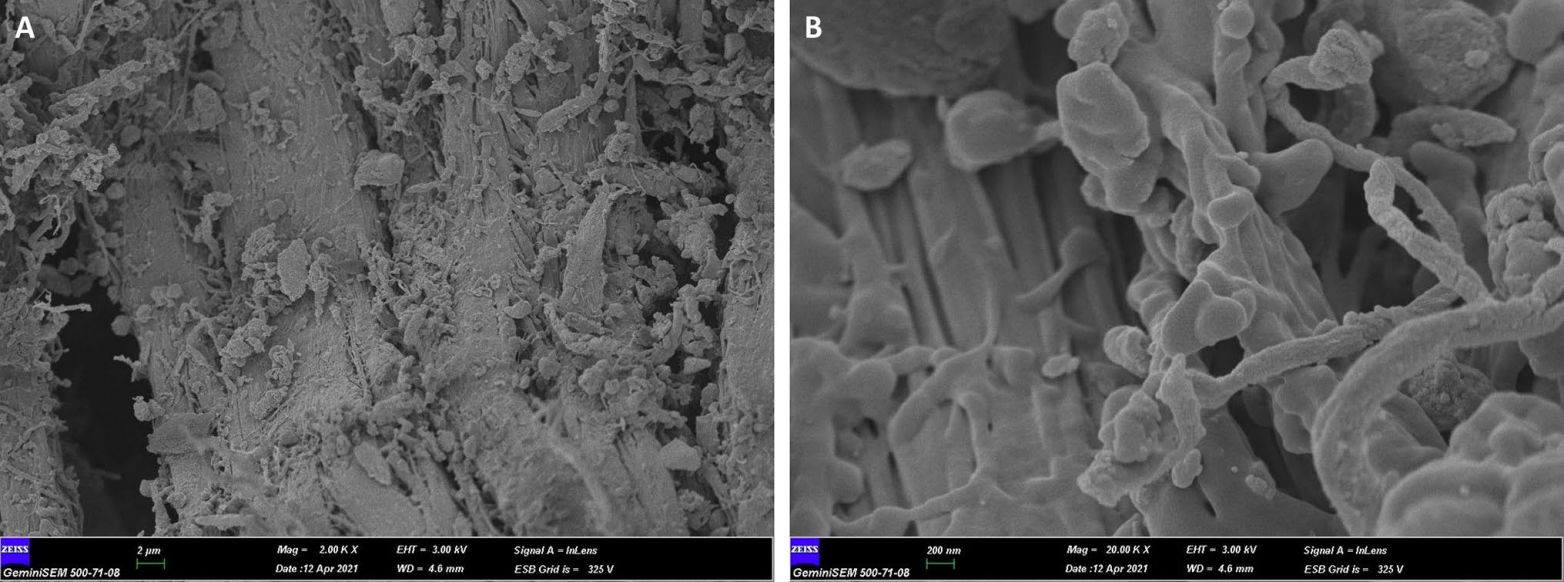
FE-SEM images of the cHAP at 2000x (**A**) and 20,000x (**B**) magnification

**Fig. 2:**
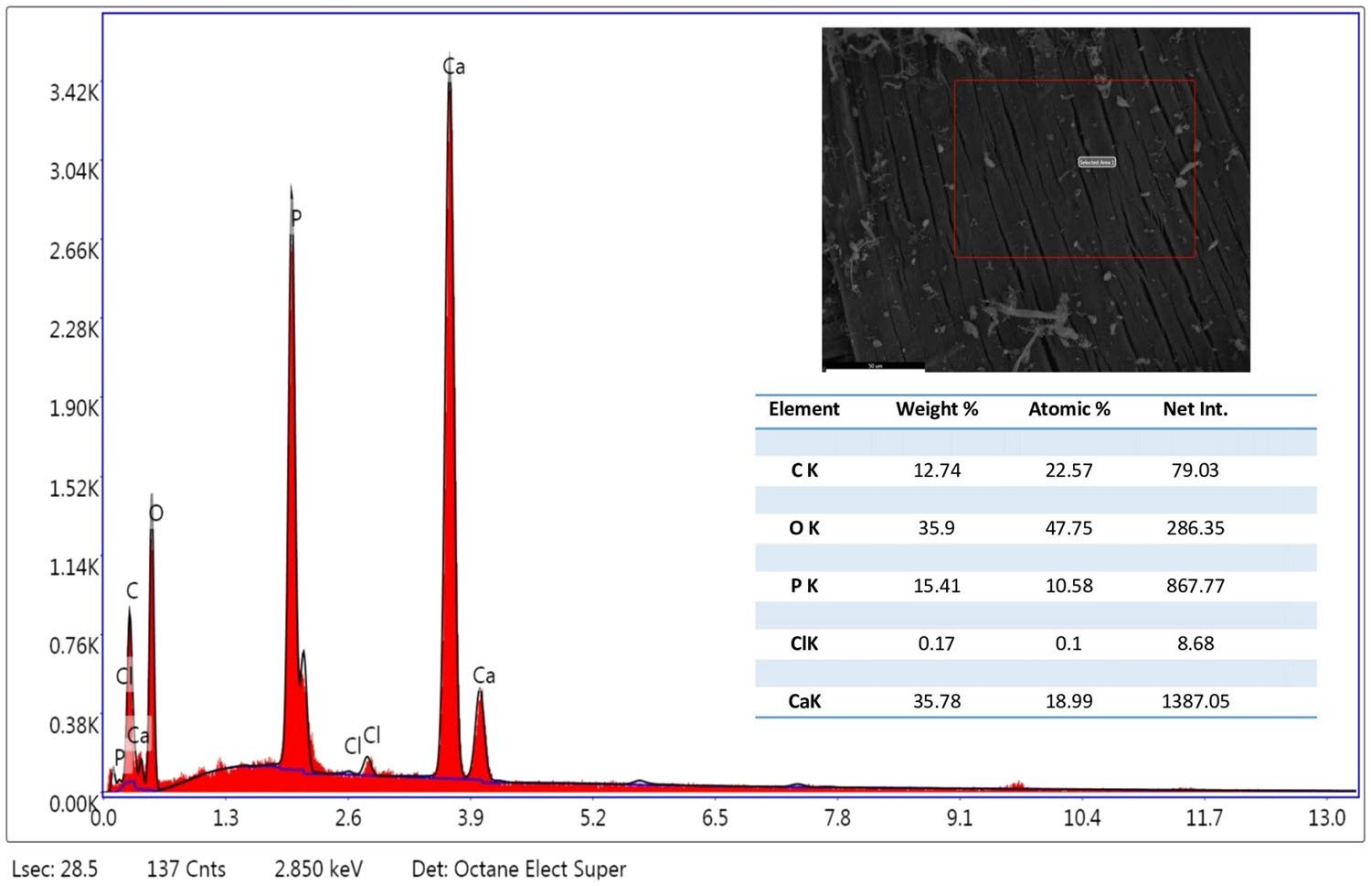
EDX spectrum of the cHAP, showing its elemental composition

**Fig. 3:**
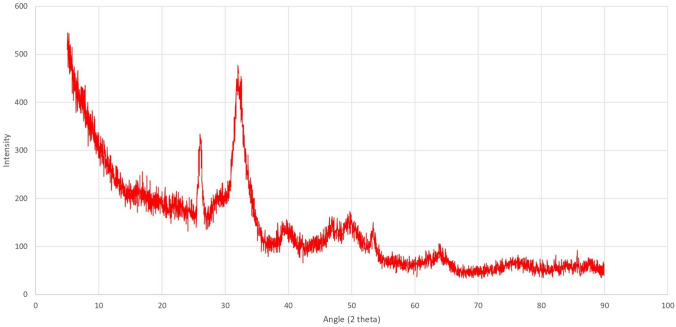
XRD pattern of the cHAP, highlighting its crystalline structure

**Fig. 4:**
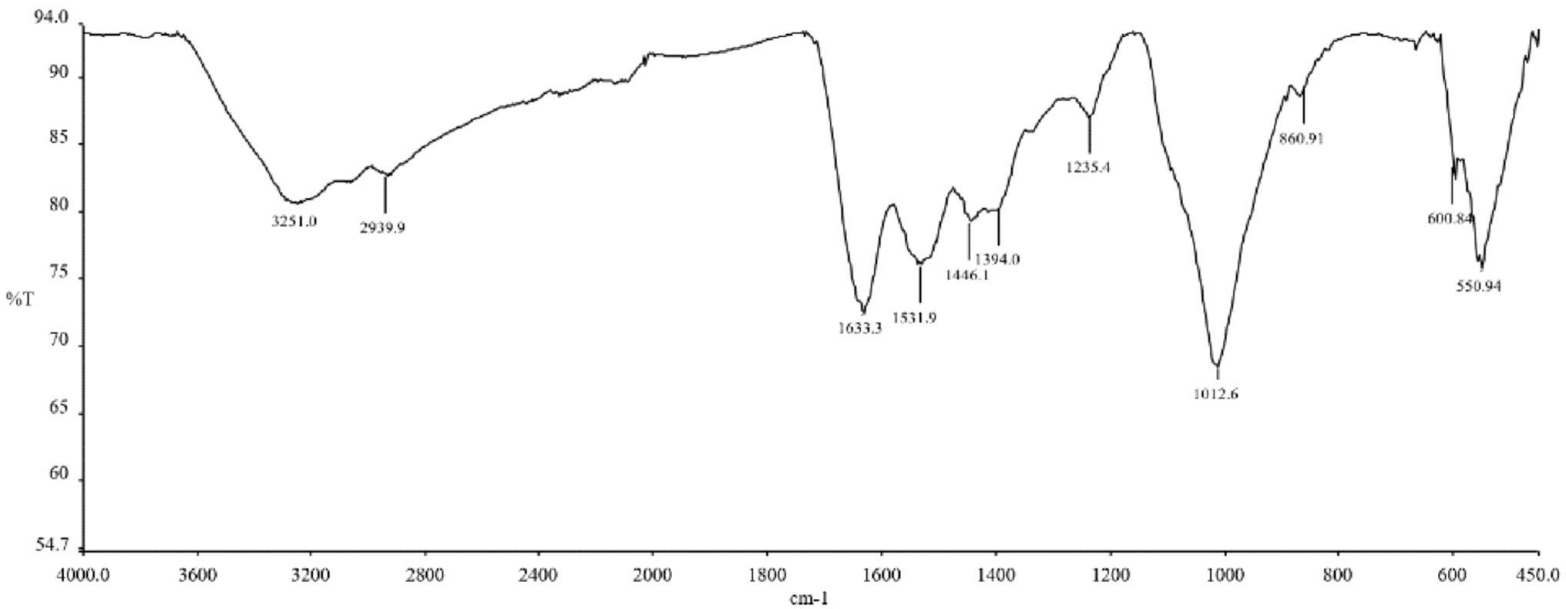
FT-IR spectrum of the cHAP showing characteristic functional group peaks

**Fig. 5:**
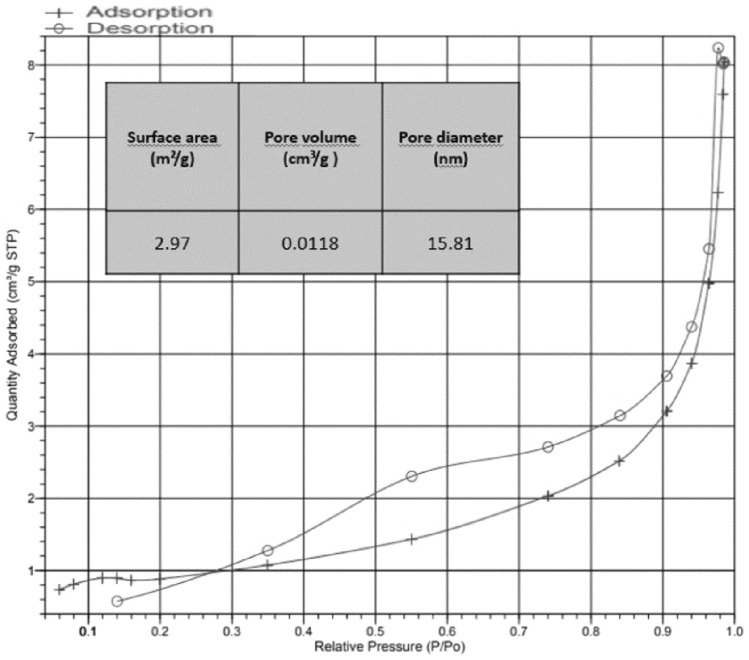
BET surface area analysis of the cHAP

The FE-SEM analysis of the cHAP nanocomposite (Fig. 1) revealed a partially heterogeneous surface morphology, reflecting the presence of both collagen and hydroxyapatite. The nanoparticle size ranged from 50 to 200 nm. Elemental distribution, assessed by SEM–EDX (Fig. 2), confirmed the presence of HAp and collagen within the nanomaterial. XRD and FT-IR spectra (Figs. 3 and 4) showed that the cHAP nanoparticles were successfully fabricated. BET results (Fig. 5) revealed an average pore volume of 0.0118 cm^3^/g and a pore diameter of 15.81 nm for the nanomaterial.

### Elemental composition of experimental pulp-capping materials

The elemental composition of the samples, as determined by EDX, is presented in Table 1. An increase in the elemental ratios of Na, Mg, P, Cl, and Ca was observed in the groups containing cHAP nanocomposite compared to the control group. Notably, the Cl content was higher in the cHAP-CHX group, while the P and Ca content remained stable in the CHX group. These findings confirm successful incorporation of both cHAP nanocomposite and CHX into the pulp-capping materials.

**Table 1:** Mean elemental content values of the samples by EDX analysis

Elements	Control	cHAP1	cHAP5	CHX	cHAP-CHX
C	37.70	35.02	37.59	36.80	38.11
O	36.55	36.19	36.35	36.49	35.67
Na	0.18	0.27	0.21	0.23	0.25
Mg	0.28	0.34	0.30	0.33	0.31
Al	2.53	2.71	2.42	2.62	2.37
Si	10.91	11.31	11.04	10.76	10.30
P	0.75	0.93	0.89	0.69	0.80
S	0.52	0.58	0.57	0.47	0.56
Cl	0.08	0.11	0.11	0.15	0.32
K	0.11	0.16	0.17	0.18	0.15
Ca	6.89	8.50	8.46	7.56	7.76
Ba	3.29	3.70	3.44	3.55	3.18
W	0.22	0.20	0.25	0.19	0.21

### Crystallinity and molecular structure of experimental pulp-capping materials

**Fig. 6:**
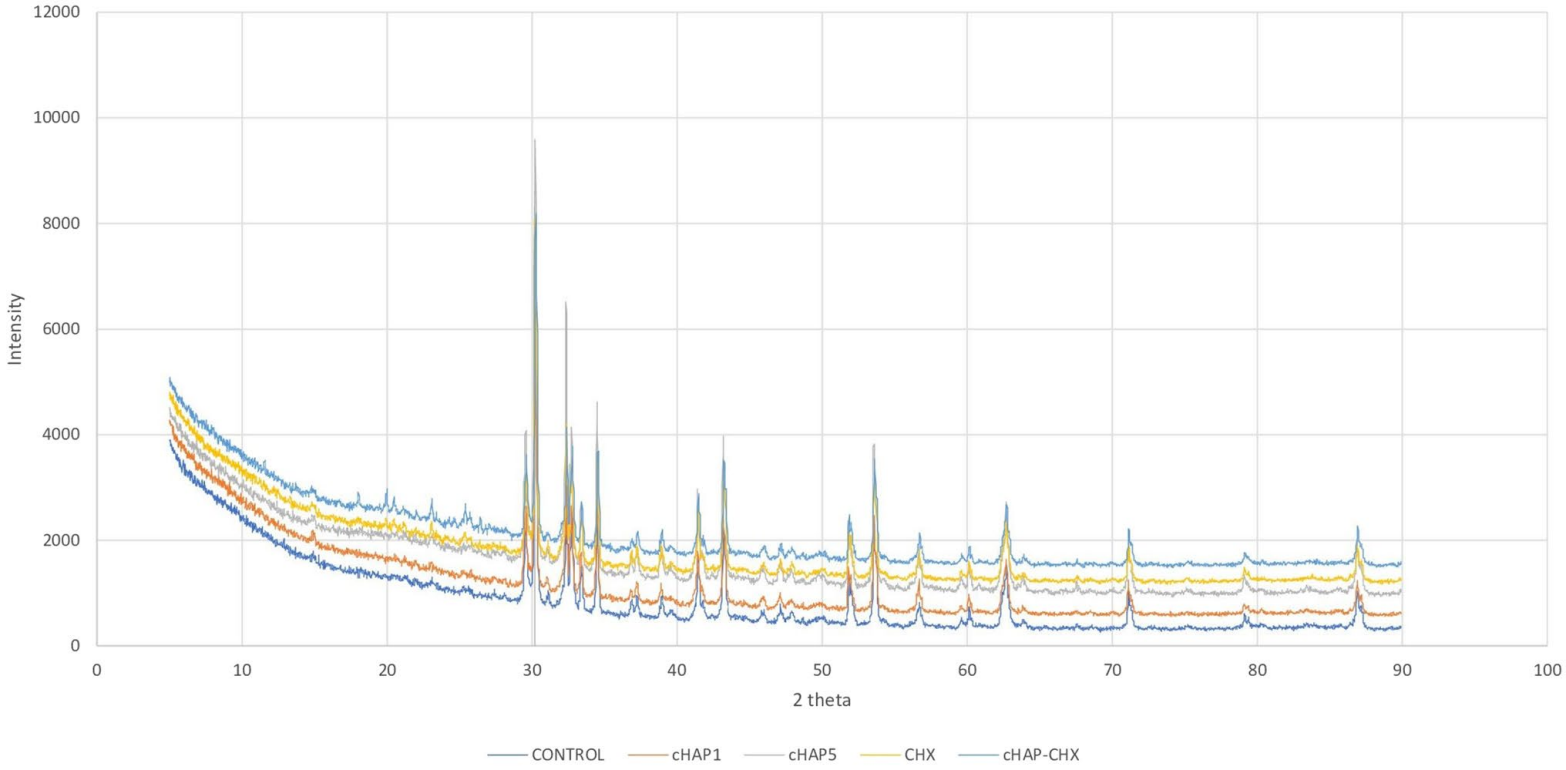
XRD patterns of the experimental pulp-capping materials. (Control: A commercially available resin-based pulp-capping material (TheraCal LC); cHAP1: Experimental resin-based pulp-capping material containing 1% cHAP; cHAP5: Experimental resin-based pulp-capping material containing 5% cHAP; CHX: Experimental resin-based pulp-capping material containing 5% chlorhexidine; cHAP-CHX: Experimental resin-based pulp-capping material containing 2.5% cHAP and 2.5% chlorhexidine)

**Fig. 7:**
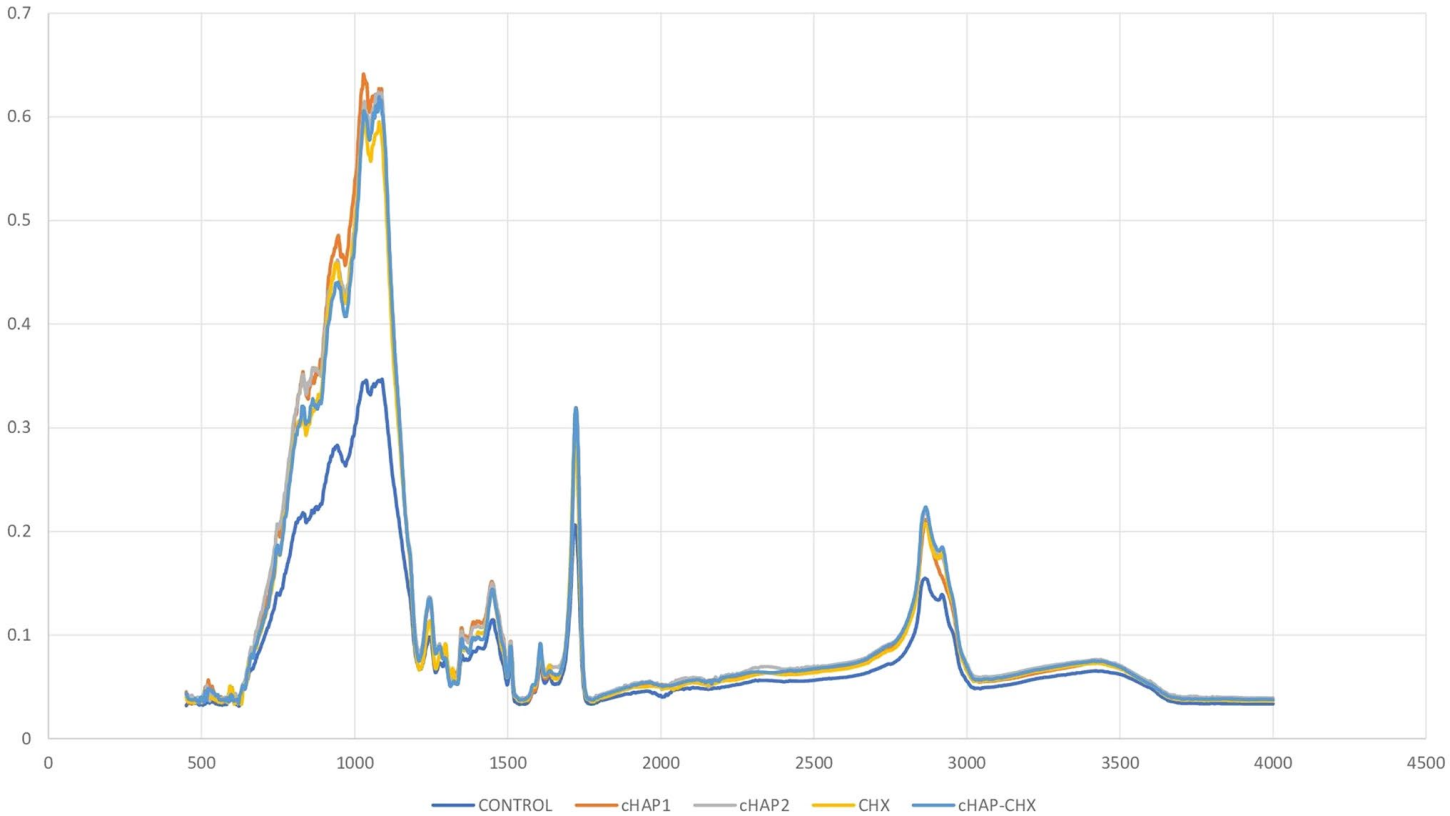
FT-IR spectra of the experimental pulp-capping materials (Control: A commercially available resin-based pulp-capping material (TheraCal LC); cHAP1: Experimental resin-based pulp-capping material containing 1% cHAP; cHAP5: Experimental resin-based pulp-capping material containing 5% cHAP; CHX: Experimental resin-based pulp-capping material containing 5% chlorhexidine; cHAP-CHX: Experimental resin-based pulp-capping material containing 2.5% cHAP and 2.5% chlorhexidine)

The XRD and FT-IR spectra of the experimental groups are shown in Figs. 6 and 7. XRD results revealed distinct peaks in the 15–30° 2θ range for CHX-containing groups, indicating successful incorporation of crystalline CHX into the pulp-capping material. The peaks from the cHAP material overlapped with the resin, due to similar composition between the cHAP extracted from fish scales and the resin-based pulp-capping material (TheraCal LC).

The FT-IR spectra showed similarities across all groups, with distinct peaks at 550 cm⁻^1^, 1014 cm⁻^1^, 1633 cm⁻^1^, and 2920 cm⁻^1^ in the cHAP-containing groups.

### Microhardness of experimental pulp-capping materials

The microhardness values of the groups are presented in Table 2. The results showed a significant reduction in microhardness only in the CHX group (*p* < 0.05). No significant difference was observed between the control group and other cHAP-containing groups (*p* > 0.05).

**Table 2:** Mean microhardness values of the groups

	Control	cHAP1	cHAP5	CHX	cHAP-CHX
MEAN	27.03^a^	28.56^a^	29.05^a^	23.71^b^	27.79^a^
SD	3.38	1.18	1.7	2.19	2.55

^*^Different letters (a, b) in the same row indicate statistically significant difference (one-way ANOVA test)

### ***D***egree of monomer conversion (DC)

The DC values for the groups are shown in Table 3. The addition of cHAP or CHX alone did not significantly affect the degree of monomer conversion compared to the control group (*p* > 0.05). However, the combination of chlorhexidine and cHAP led to a significant increase in the degree of monomer conversion (*p* < 0.05).

**Table 3:** Degree of monomer conversion (DC) values of the groups

	Control	cHAP1	cHAP5	CHX	cHAP-CHX
MEAN	41.17^a^	46.09^a,b^	44.45^a,b^	43.75^a,b^	49.44^b^
SD	5.35	5.86	6.14	7.75	7.98

^*^Different letters (a, b) in the same row indicate statistically significant difference (one-way ANOVA test)

### Antibacterial activity

**Fig. 8:**
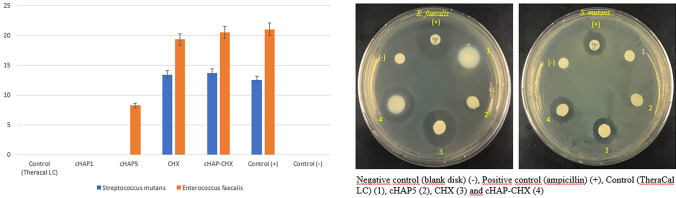
Antimicrobial activity of the experimental pulp-capping materials against *E. faecalis* and *S. mutans* strains. (Negative control: Bblank disk) (−), Positive control (Ampicillin) ( +), Control (TheraCal LC) (1), cHAP5 (2), CHX (3) and cHAP-CHX (4). (Control: A commercially available resin-based pulp-capping material (TheraCal LC); cHAP1: Experimental resin-based pulp-capping material containing 1% cHAP; cHAP5: Experimental resin-based pulp-capping material containing 5% cHAP; CHX: Experimental resin-based pulp-capping material containing 5% chlorhexidine; cHAP-CHX: Experimental resin-based pulp-capping material containing 2.5% cHAP and 2.5% chlorhexidine)

The antimicrobial activities of the Control (TheraCal LC), cHAP1, cHAP5, CHX, and cHAP-CHX materials against the test microorganisms (*E. faecalis* and *S. mutans*) are presented in Table 4 and Fig. 8. The results confirmed antibacterial activity of cHAP, CHX, and cHAP-CHX against these bacteria.

**Table 4:** Inhibition zone diameters (mm) formed by the pulp-capping material against microorganisms (*S. mutans* and *E. feacalis*) tested by the agar diffusion method

	Control (Theracal LC)	cHAP1	cHAP5	CHX	cHAP-CHX	Control (−)	Control ( +)
*E. feacalis*	0	0	8.25^a^	19.3^b^	20.5^b^	0	21.0^b^
*S. mutans*	0	0	0	13.4^a^	13.7^a^	0	12.5^a^

^*^Different letters in the same row indicate statistical difference (one-way ANOVA test)

The Control group (TheraCal LC) did not show any inhibition zones against *E. faecalis* or *S. mutans*, indicating a lack of antimicrobial effect. The cHAP5 group exhibited antibacterial activity only against *E. faecalis*, with an inhibition zone diameter of 8.25 mm. CHX demonstrated antimicrobial activity against both *E. faecalis* and *S. mutans,* with inhibition zone diameters of 19.3 mm and 13.4 mm, respectively. The cHAP-CHX group also displayed antimicrobial activity against both *E. faecalis* and *S. mutans*, with inhibition zone diameters of 20.5 mm and 13.7 mm, respectively (Table 4). The inhibition zones for the test microorganisms ranged from 8.25 mm to 20.5 mm. *E. faecalis* was identified as the most sensitive strain against the tested pulp-capping materials*,* with cHAP-CHX showing the highest antimicrobial activity (20.5 mm). These findings suggest that the combination of cHAP and chlorhexidine significantly enhances the antibacterial properties of the pulp-capping material, offering greater protection against microbial contamination in dental pulp treatments.

When evaluating the mean inhibition zones against *E. faecalis*, the CHX and cHAP-CHX groups exhibited significantly greater antibacterial activity than the Control (TheraCal LC), cHAP1, and cHAP5 groups (*p* < 0.05). No significant difference was observed between the Control ( +) group (Ampicillin) and the CHX or cHAP-CHX groups (*p* > 0.05). The cHAP5 group demonstrated significantly greater antibacterial activity against *E. faecalis* compared to the Control (TheraCal LC) and cHAP1 groups (*p* < 0.05).

Similarly, for *S. mutans*, the CHX and cHAP-CHX groups again exhibited significantly greater antibacterial activity compared to the Control (TheraCal LC), cHAP1, and cHAP5 groups (*p* < 0.05). No significant difference was found between the Control ( +) group (Ampicillin) and the CHX or cHAP-CHX groups (*p* > 0.05).

## Discussion

This study aimed to develop an experimental pulp-capping material by incorporating a collagen–hydroxyapatite nanocomposite (cHAP) derived from fish scales, with chlorhexidine (CHX) added as an antimicrobial agent. The material was evaluated for microhardness, degree of monomer conversion (DC), and antibacterial activity.

The addition of cHAP, either alone or in combination with CHX, numerically increased the microhardness of the pulp-capping material. However, the increase was not statistically significant compared to the control group. This suggests that while cHAP may influence the physical properties of the material, its effect on microhardness is minimal. Consequently, the first hypothesis of this study, which proposed that the addition of cHAP would increase the microhardness of the material, was rejected. The degree of monomer conversion, which reflects polymerization efficiency, was unaffected by cHAP alone. However, a notable improvement was observed when cHAP was combined with CHX. This suggests that CHX may enhance polymerization of the material. Consequently, the second hypothesis, which posited that cHAP would improve DC, was also rejected, as the material's conversion was only enhanced when both cHAP and CHX were present. As for the antibacterial properties, the results showed that the incorporation of cHAP, either alone or with CHX, improved the antibacterial activity of the material, particularly against *E. faecalis*. The combination of cHAP with CHX demonstrated significant antibacterial activity against both *E. faecalis* and *S. mutans*, while cHAP alone showed efficacy only against *E. faecalis*. These findings support the third hypothesis, which proposed that the inclusion of cHAP, either with or without CHX, would enhance the antibacterial properties of the material. However, since the antibacterial effect was more pronounced when both components (cHAP and CHX) were combined, the hypothesis was partially accepted.

Previous studies have shown that fish scales contain various elements, including Na, P, Cl, and Ca [27, 28]. EDX analysis conducted in our study confirmed the presence of these elements. The EDX analysis also demonstrated that the Cl ratio of the groups containing CHX was high, as expected. According to studies involving XRD, CHX has a crystalline structure, showing peaks in the 2θ range of 15–30° [29], which was also observed in the current study. This consistency with prior findings confirms the successful inclusion of CHX in the material matrix. The XRD peaks associated with the fish scale-derived HAp overlapped with the peaks from the pulp-capping material used (TheraCal LC), indicating that the cHAP component did not introduce new crystalline phases that could disrupt the overall structure of the pulp-capping material. Instead, it contributed to a more integrated composite structure.

Similarly, the FT-IR spectra of the experimental groups containing both CHX and cHAP exhibited a pattern similar to that of the control group. However, distinct peaks were observed in the samples containing cHAP, particularly at specific wavelengths associated with collagen and hydroxyapatite components (e.g., peaks around 550 cm^−1^, 1014 cm^−1^, 1633 cm^−1^, and 2920 cm^−1^). These findings suggest that while the incorporation of CHX and cHAP did not dramatically alter the overall molecular structure, it influenced the characteristics of the composite material, as evidenced by shifts in the intensity of certain peaks. This is an important indication that the composite material retained its essential characteristics while incorporating antimicrobial properties from CHX.

Theracal LC, a light-curing resin-modified calcium silicate-based capping material, was utilized in this study. As with all resin-based materials, some residual monomers may remain due to incomplete polymerization [30]. These residual monomers can potentially cause pulp irritation, highlighting the importance of high polymerization rates in resin-based materials to minimize the presence of unreacted monomers. Ensuring efficient polymerization is crucial for the success of resin-based pulp-capping agents, as it directly impacts the material's biocompatibility and performance. Previous studies have reported DC values for resin-based pulp-capping materials ranging from 10 to 40%, depending on sample thickness [31, 32]. In the present study, the DC values of the groups ranged from 40 to 50%, indicating a more efficient polymerization process. The DC was significantly increased in the cHAP-CHX group. This improvement in monomer conversion may be explained by enhanced light absorption during the light-curing process, potentially due to an increase in the crystallinity of the material. The crystalline structure of hydroxyapatite and CHX may have increased the material's ability to absorb light more effectively during the light-curing process, leading to a more efficient polymerization by allowing greater light penetration. This result aligns with the previous findings [26]. The improved monomer conversion observed in the cHAP-CHX group suggests that the addition of cHAP and CHX did not impair the polymerization properties of the material. Instead, their combined effects may have enhanced the overall performance of the pulp-capping material, promoting better conversion of monomers into polymerized material and potentially reducing the occurrence of residual monomers that could cause pulp irritation.

The Vickers hardness test results revealed that the addition of CHX alone to the capping material significantly reduced its microhardness. This decrease may be attributed to the softer nature of CHX particles compared to the resin-based material. Chlorhexidine, as a relatively soft substance, likely did not contribute to strengthening the material matrix, leading to a reduction in the overall hardness. This observation is consistent with the previous research [26]. In contrast, the addition of cHAP, either alone or in combination with CHX, did not significantly affect the microhardness of the material. This could be due to the low percentage of functional components (cHAP and CHX) in the formulation, which may not have been sufficient to substantially affect the overall microhardness of the material. Additionally, the structural similarity between cHAP and the pulp-capping material may have contributed to the lack of significant change in hardness.

An ideal pulp capping material should exhibit antibacterial activity, as this is essential for protecting the dental pulp from infection and promoting healing. Calcium silicate-based materials are known to have better antimicrobial activity than calcium hydroxide-based materials [33]. Hydraulic calcium silicate-based materials (HCSMs) gradually release hydroxyl ions, leading to a sustained alkalinization, which contributes to prolonged antimicrobial action while minimizing abrupt pH shifts that could damage surrounding tissues. In contrast, Ca(OH)₂ raises the pH rapidly but loses its effectiveness over time as it dissolves or washes out [33, 34]. The antimicrobial effectiveness of each material varies depending on factors, such as the microorganism targeted, duration of exposure, and the amount of drug released. The antibacterial mechanism of hydraulic calcium silicate cements is attributed to the release of calcium ions (Ca^2+^) and hydroxyl ions (OH^−^) during hydration, which increases the material’s alkalinity [35, 36]. In the current study, the antibacterial activity of the materials was tested against *S. mutans*, a caries-associated bacterium, and *E. feacalis*, a microorganism commonly detected in endodontic infections. The collagen–hydroxyapatite nanocomposite derived from fish scales was incorporated into the material’s composition, considering that it could improve the antibacterial and mineralizing properties of the material by increasing the release of calcium and hydroxyl ions, thereby improving its alkanity.

Although previous studies have reported the antimicrobial effectiveness of TheraCal LC [31, 33], it did not create inhibition zones against *E. faecalis* and *S. mutans* in the present study, indicating a lack of antimicrobial activity of this material against these microorganisms. This discrepancy could be explained by differences in the antibacterial testing methods or the bacterial strains used. Our study also found that the addition of cHAP at a low concentration (1%) did not enhance the antibacterial activity of the material. However, when the cHAP concentration was increased, antibacterial activity against *E. feacalis* improved, though there was no significant effect on *S. mutans*. This finding supports the aforementioned argument that the antibacterial activity of a material may vary depending on the microorganism and concentration. The highest antibacterial activity was observed when CHX, a well-known broad-spectrum antibacterial agent, was added to the material alone or in combination with cHAP. Thus, an experimental pulp-capping material with antimicrobial activity against both *E. feacalis* and *S. mutans* was achieved.

Chlorhexidine diacetate is a soluble compound, and its incorporation into materials alone may compromise mechanical strength over time. However, when combined with cHAP, this potential drawback can be mitigated, enhancing the material’s overall properties. Based on the results of the present study, the resin-based pulp-capping material containing both CHX and cHAP nanostructure derived from fish scales demonstrated the most favorable balance of properties among the tested materials. Several studies have investigated the impact of chlorhexidine (CHX) on the degree of monomer conversion (DC) in dental materials, yielding varied results. One study examined the effect of CHX content on DC and E-modulus in experimental adhesive blends. The findings indicated that increasing concentrations of CHX had minimal adverse effects on DC but decreased the E-modulus by 27–48% compared to controls [37]. Another study evaluated the antimicrobial activity, DC, and Knoop hardness (KH) of experimental infiltrants incorporating CHX. The study concluded that DC was not affected by the monomer blend composition or CHX concentration [38]. A study investigated the effect of various CHX concentrations on the DC of an experimental adhesive resin. The results showed that 1% CHX did not significantly alter the DC, while 5% CHX led to a significant decrease in DC [39]. In brief, the impact of CHX on DC varies depending on its concentration and the specific dental material involved. While some studies report minimal effects, others indicate a reduction in DC with higher CHX concentrations. Therefore, incorporating CHX into dental materials requires careful consideration of its concentration to balance antimicrobial benefits with potential alterations in polymerization efficiency.

In summary, the findings of this study suggest that incorporating fish scale-derived cHAP and CHX into pulp-capping materials holds promise for developing novel materials with enhanced antibacterial effectiveness. However, as this was an in vitro study, several factors, such as saliva in the oral cavity, chewing forces, intraoral thermal and pH changes, and the effect of pulpal responses, were not accounted for. Additionally, in this study, the properties of a resin-based calcium silicate material were evaluated after the addition of cHAP and CHX, but no comparison was made with other commercially available pulp-capping materials (such as MTA or calcium hydroxide). Further in vivo and in vitro studies are warranted to validate these findings and confirm their clinical relevance.

## Conclusion

Within the limitations of this study, the addition of chlorhexidine (CHX) alone to the pulp-capping material was found to reduce its microhardness, potentially compromising its mechanical stability. Conversely, the incorporation of fish scale-derived calcium hydroxyapatite (cHAP) combined with CHX significantly increased the degree of monomer conversion, enhancing the polymerization process. The inclusion of cHAP, either alone or in combination with CHX, improved the material's antibacterial efficacy against *E. faecalis*. Notably, the combination of cHAP and CHX exhibited superior antibacterial activity against *S. mutans*.

Overall, materials containing CHX alone or in combination with cHAP exhibited the strongest antimicrobial effects against both bacterial strains, highlighting their potential to improve the performance of pulp-capping materials. The integration of cHAP and chlorhexidine in resin-based pulp-capping materials offers a promising approach for enhancing both antibacterial activity and biocompatibility, presenting a potential advancement for pulp-capping procedures in clinical practice.

## Data Availability

Data are available on request from the authors.
